# Evaluation of Clusterin, Sestrin-2, Oxidative Stress, and Inflammatory Indices in Patients with Alzheimer’s Disease Dementia

**DOI:** 10.3390/brainsci16090977

**Published:** 2026-09-15

**Authors:** Nida Aslan Karakelle, Safiye Göçer, Esra Eruyar, Yasemin Atıcı, Müge Coşkun Yıldırım

**Affiliations:** 1 Department of Physiology, Faculty of Medicine, Lokman Hekim University, 06510 Ankara, Turkey; 2 Department of Medical Microbiology, Faculty of Medicine, Lokman Hekim University, 06510 Ankara, Turkey; safiyegocer@lokmanhekim.edu.tr; 3 Department of Neurology, Faculty of Medicine, Lokman Hekim University, 06510 Ankara, Turkey; dr.esrayetkin@gmail.com; 4 Department of Medical Biochemistry, Faculty of Medicine, Lokman Hekim University, 06510 Ankara, Turkey; yasemin.atici@lokmanhekim.edu.tr; 5 Department of Biostatistics, Faculty of Medicine, Hacettepe University, 06800 Ankara, Turkey; mugecoskun96@gmail.com

**Keywords:** dementia, oxidative stress, clusterin, sestrin, inflammation index

## Abstract

**Highlights:**

**What are the main findings?**

- Alzheimer’s disease dementia was associated with increased oxidative stress.
- Clusterin increased, whereas sestrin-2 decreased, in Alzheimer’s disease dementia.

**What are the implications of the main findings?**

- Clusterin and sestrin-2 may contribute to Alzheimer’s disease dementia pathophysiology.
- These biomarkers may have potential relevance as candidate diagnostic markers.

**Abstract:**

**Introduction:** Alzheimer’s disease dementia (AD dementia) is a progressive neurodegenerative disorder associated with cognitive decline. Oxidative stress and inflammation may contribute to its pathophysiology, with clusterin (CLU) and sestrin-2 (SESN2) emerging as potential biomarkers. This study evaluated oxidative stress markers, inflammatory indices, and serum CLU and SESN2 levels in patients with AD dementia. **Methods:** A total of 48 patients with AD dementia and 39 healthy controls in a broadly comparable age range were enrolled from a neurology outpatient clinic. AD dementia was diagnosed according to internationally accepted clinical criteria. Cognitive function was assessed using Mini-Mental State Examination (MMSE) scores obtained at diagnosis. Serum malondialdehyde (MDA), glutathione (GSH), CLU, and SESN2 were measured, with MDA and GSH analyzed spectrophotometrically and CLU and SESN2 concentrations determined using the enzyme-linked immunosorbent assay (ELISA). Inflammatory indices were calculated from routine hematological and biochemical parameters. **Results:** A multivariable logistic regression model including age, MDA, and GSH showed excellent discriminatory performance for distinguishing patients with AD dementia from healthy controls. Patients with AD dementia had significantly lower SESN2 levels (*p* < 0.001) and higher CLU levels (*p* < 0.001) than controls. No significant associations were observed between inflammatory indices and the investigated clinical or biochemical variables in the AD dementia group (*p* > 0.05). **Conclusions:** Oxidative stress and altered SESN2 and CLU levels may be associated with AD dementia, whereas the inflammatory indices showed limited utility. Larger prospective studies are needed to validate these findings and clarify their clinical significance.

## 1. Introduction

Dementia is a neurodegenerative syndrome characterized by progressive deterioration of cognitive functions that significantly impairs an individual’s ability to perform activities of daily living independently. The term dementia, commonly referred to as “senility” or “loss of cognitive function” in the general population, is derived from the Latin word mens (mind) and literally means “loss of mind” [1]. With increasing life expectancy and the rapid growth of the aging population worldwide, the global prevalence of dementia has risen substantially, imposing a considerable burden on affected individuals, caregivers, healthcare systems, and society. Consequently, the World Health Organization has recognized dementia as one of the major global public health priorities due to its profound medical, social, and economic consequences [2].

Alzheimer’s disease (AD) is the most common cause of dementia, accounting for approximately 60–70% of all dementia cases. The pathogenesis of AD is multifactorial and involves a complex interplay of aging, genetic susceptibility, vascular dysfunction, neuroinflammation, oxidative stress, and other pathogenic mechanisms. Although advanced age and female sex are recognized as the strongest risk factors, accumulating evidence indicates that environmental and lifestyle-related factors, including low educational attainment, cigarette smoking, vitamin deficiencies, chronic inflammation, and oxidative damage, also contribute to disease development. Therefore, identifying the early biological alterations associated with AD and discovering reliable biomarkers are of paramount importance for improving early diagnosis and developing targeted therapeutic strategies [3,4]. Oxidative stress is currently considered one of the principal pathological mechanisms underlying AD and other forms of dementia. Owing to its high oxygen consumption, abundant lipid content, and relatively limited antioxidant defense capacity, the brain is particularly vulnerable to oxidative damage induced by reactive oxygen species (ROS). Excessive oxidative stress promotes lipid peroxidation, protein oxidation, and DNA damage, thereby accelerating neuronal dysfunction and loss. Moreover, oxidative stress contributes to the accumulation of amyloid-β (Aβ) peptides and the formation of neurofibrillary tangles, which are the neuropathological hallmarks of AD [5,6]. In addition to oxidative stress, neuroinflammation has emerged as a critical contributor to the initiation and progression of neurodegenerative diseases. In recent years, systemic inflammatory indices derived from peripheral blood cell counts have gained considerable attention as practical, inexpensive, and readily available biomarkers for assessing the inflammatory burden associated with neurodegeneration. Among these, the neutrophil-to-lymphocyte ratio (NLR), platelet-to-lymphocyte ratio (PLR), systemic inflammation response index (SIRI), aggregate index of systemic inflammation (AISI), and systemic immune–inflammation index (SII) have demonstrated potential clinical utility in the diagnosis, assessment of disease severity, and prognostic evaluation of patients with Alzheimer’s disease and other dementias [7,8].

CLU, also known as apolipoprotein J (ApoJ), has emerged as one of the most important genetic susceptibility factors associated with Alzheimer’s disease (AD). First isolated from testicular tissue in 1983, this heterodimeric glycoprotein was named ‘CLU’ because of its ability to promote cell aggregation [9]. CLU functions as an extracellular molecular chaperone by binding to the exposed hydrophobic regions of misfolded proteins under conditions of cellular stress, thereby preventing their aggregation. In addition, CLU has been shown to interact with Aβ peptides, modulating fibril formation and influencing amyloid deposition, one of the principal neuropathological hallmarks of AD [10]. Beyond its chaperone activity, intracellular CLU has been implicated in the regulation of apoptosis, inflammatory signaling, and oxidative stress responses, suggesting a protective role in neuronal survival under pathological conditions [11,12]. More recently, accumulating evidence has indicated that CLU may also facilitate the propagation of tau pathology, another key feature of AD [13]. Supporting this hypothesis, an experimental animal study demonstrated that intracerebral administration of CLU-containing medium into the rat hippocampus resulted in increased tau levels in CLU-treated animals [14]. Furthermore, CLU has been identified as a key molecule involved in neuroprotective responses and is increasingly recognized as one of the major pathological biomarkers associated with AD [15,16]. A longitudinal cohort study involving individuals with mild cognitive impairment (MCI) reported that cerebrospinal fluid (CSF) CLU concentrations were significantly associated with established AD biomarkers. The authors further suggested that CLU may serve as a potential neuroprotective factor capable of preserving cognitive function during the early stages of cognitive decline [17]. Collectively, these findings highlight CLU as a promising candidate for improving our understanding of AD pathogenesis and for the development of novel diagnostic biomarkers and therapeutic targets.

Another family of proteins that has attracted considerable attention in neurodegenerative diseases is the SESN family. Comprising three members, SESN1, SESN2, and SESN3, these stress-inducible proteins play pivotal roles in cellular defense mechanisms against oxidative stress and genotoxic damage [18,19]. Among them, SESN2 has emerged as a key regulator of antioxidant defense, autophagy, and metabolic homeostasis. Experimental studies have demonstrated that Aβ exposure upregulates SESN2 expression in primary cortical neurons, thereby enhancing antioxidant responses and promoting autophagic activity. Furthermore, SESN2 has been reported to exert neuroprotective effects by scavenging ROS, facilitating mitophagy to eliminate damaged mitochondria, and coordinating cellular stress-response pathways [20]. In contrast, excessive oxidative stress activates p53- and FOXO-mediated apoptotic signaling pathways, whereas mild or transient oxidative stress induces SESN expression, thereby suppressing apoptosis and enhancing cellular adaptation to stress [21,22]. Collectively, these findings suggest that SESNs, particularly SESN2, may represent promising biomarkers and potential therapeutic targets for Alzheimer’s disease and other neurodegenerative disorders due to their critical roles in maintaining redox homeostasis and promoting neuronal survival.

Despite growing evidence implicating oxidative stress, inflammation, and cellular stress-response pathways in the pathogenesis of dementia, studies comprehensively evaluating these mechanisms in an integrated manner remain limited. Therefore, the present study aimed to investigate serum CLU and SESN2 levels, together with the oxidative stress markers MDA and reduced GSH, as well as systemic immune–inflammatory indices, in patients with dementia. In addition, the relationships between these laboratory parameters and the demographic and clinical characteristics of the study population were examined. We hypothesized that the combined assessment of these biomarkers would provide further insight into the interplay between oxidative stress, inflammation, and cellular stress responses in AD dementia. The findings of this study may contribute to the identification of novel biomarkers associated with AD dementia and provide valuable evidence to support early diagnosis, risk stratification, and the development of targeted therapeutic strategies.

## 2. Materials and Methods

### 2.1. Study Design

This retrospective observational study included 48 patients with AD dementia who attended the neurology outpatient clinic of Lokman Hekim Akay Hospital between August 2022 and July 2024, along with a healthy control group in a broadly comparable age range. The median age of the AD dementia group was 78.5 years (range, 65–89 years), whereas the median age of the control group was 67 years (range, 59–85 years). The study protocol was approved by the Ethics Committee of Lokman Hekim University (approval no. 2022110; approval date: 3 August 2022), and all study procedures were conducted in accordance with the ethical principles of the Declaration of Helsinki. Medical records of patients diagnosed with AD dementia and followed at our institution were reviewed retrospectively. Only patients with late-onset Alzheimer’s disease (AD) dementia were included in the study. AD dementia was defined as symptom onset at or after 65 years of age. The diagnosis of AD dementia was established according to internationally accepted diagnostic criteria based on comprehensive clinical assessment, neuropsychological evaluation, and neuroimaging findings when clinically indicated. Cognitive function and dementia severity were assessed using the MMSE, and the MMSE scores obtained at the time of diagnosis were included in the statistical analyses. To improve the homogeneity of the study population and minimize potential confounding factors, patients with a history of malignancy, early-onset Alzheimer’s disease (age at symptom onset <65 years), traumatic brain injury, or dementia subtypes other than Alzheimer’s disease including vascular dementia, dementia with Lewy bodies, frontotemporal dementia, and other secondary causes of dementia were excluded. Demographic characteristics, clinical findings, laboratory parameters, and MMSE scores of all eligible participants were retrieved from the electronic medical record system and included in the statistical analyses.

### 2.2. Sample Collection and Biochemical Analyses

Fasting venous blood samples were collected from all participants after an overnight fast of at least 8 h. Blood samples were obtained in the morning using serum separator tubes (Vacutainer SST, Becton Dickinson, Franklin Lakes, NJ, USA) and centrifuged at 1300× *g* for 10 min, and the separated serum was transferred into Eppendorf tubes and stored at −80 °C until biochemical analysis. For hematological analyses, whole blood samples were collected into K2-EDTA tubes (Vacuette^®^, 3 mL K2 EDTA; Greiner Bio-One, Kremsmünster, Austria) and analyzed using a Sysmex XN-1000 automated hematology analyzer (Sysmex Corporation, Kobe, Japan). Complete blood count parameters were used to calculate the systemic immune–inflammatory indices, including the systemic immune–inflammation index (SII), aggregate index of systemic inflammation (AISI), systemic inflammation response index (SIRI), lymphocyte-to-monocyte ratio (LMR), platelet-to-lymphocyte ratio (PLR), neutrophil-to-lymphocyte ratio (NLR), derived neutrophil-to-lymphocyte ratio (dNLR), and lymphocyte-to-C-reactive protein ratio (LCR). For biochemical analyses, serum samples were collected in gel separator tubes (Vacuette^®^, 5 mL Serum Gel Tube; Greiner Bio-One, Kremsmünster, Austria) and analyzed immediately after centrifugation or following storage under appropriate conditions. Serum ferritin concentrations were measured using an electrochemiluminescence immunoassay (ECLIA) on a Cobas e601 analyzer (Roche Diagnostics, Basel, Switzerland). Serum C-reactive protein (CRP) levels were determined by a turbidimetric assay using a Cobas c501 analyzer (Roche Diagnostics, Basel, Switzerland). Additional biochemical parameters, including fasting blood glucose, urea, creatinine (CR), estimated glomerular filtration rate (eGFR), hemoglobin, vitamin B12, vitamin D, folate, high-density lipoprotein cholesterol (HDL-C), low-density lipoprotein cholesterol (LDL-C), triglycerides, alanine aminotransferase (ALT), aspartate aminotransferase (AST), gamma-glutamyl transferase (GGT), and thyroid-stimulating hormone (TSH), were measured using standard photometric methods on the Cobas c501 chemistry analyzer (Roche Diagnostics, Basel, Switzerland).

### 2.3. Measurement of Serum CLU and SESN2 Concentrations

Serum CLU and SESN2 concentrations were measured using commercially available sandwich enzyme-linked immunosorbent assay (ELISA) kits according to the manufacturers’ instructions. Serum CLU levels were determined using a human CLU ELISA kit (Elabscience, Catalog No. E-EL-H0038, Houston, Texas, USA), while serum SESN2 levels were measured using a human SESN2 ELISA kit (ELK Biotechnology, Catalog No. ELK4979, Sugar Land, Texas, USA). The intra-assay and inter-assay coefficients of variation were <8% and <10%, respectively, as reported by the manufacturers. Standard solutions supplied with each kit were serially diluted according to the manufacturer’s protocol to generate calibration curves covering the specified concentration ranges. Serum concentrations of CLU and SESN2 were calculated from the corresponding standard curves. The analytical detection ranges were 1.56–100 ng/mL for CLU and 0.16–10 ng/mL for SESN2.

### 2.4. Measurement of Serum MDA and GSH Concentrations

Serum malondialdehyde (MDA) concentrations were determined using the thiobarbituric acid-reactive substances (TBARS) method, based on the procedure described by Draper and Hadley [23]. Briefly, MDA in the serum samples was allowed to react with thiobarbituric acid under acidic conditions, resulting in the formation of a pink-colored MDA–thiobarbituric acid adduct. Following the reaction, the absorbance of the samples was measured spectrophotometrically at 532 nm. MDA concentrations were calculated using the appropriate calibration curve/formula and are expressed as µmol/L.

Reduced glutathione (GSH) concentrations were determined using the colorimetric 5,5′-dithiobis-(2-nitrobenzoic acid) (DTNB) method described by Ellman [24]. In this assay, GSH reacts with DTNB to form the yellow-colored 5-thio-2-nitrobenzoic acid (TNB), whose absorbance is measured at 412 nm. Serum samples were reacted with DTNB under the specified assay conditions, and the resulting absorbance was recorded spectrophotometrically at 412 nm. GSH concentrations were calculated using the corresponding calibration curve/formula and are expressed as µmol/L.

### 2.5. Statistical Analysis

Statistical analyses were performed using IBM SPSS Statistics for Windows, Version 29.0 (IBM Corp., Armonk, NY, USA). The normality of continuous variables was evaluated using the Shapiro–Wilk test and visual inspection of quantile–quantile (Q–Q) plots. Continuous variables with a normal distribution are expressed as mean ± standard deviation (SD), whereas non-normally distributed variables are presented as median (minimum–maximum). Categorical variables were summarized as frequencies and percentages. Comparisons between the AD dementia and control groups were performed using the independent-samples Student’s *t*-test for normally distributed continuous variables and the Mann–Whitney *U* test for variables that were not normally distributed. Categorical variables were compared using Pearson’s chi-square test. Binary logistic regression analysis was performed to identify independent factors associated with AD dementia. Receiver operating characteristic (ROC) curve analysis was used to evaluate the diagnostic performance of MDA, reduced GSH, and age, and to determine their optimal cut-off values for discriminating patients with AD dementia from healthy controls. A two-tailed *p*-value < 0.05 was considered statistically significant. In addition, graphical figures were generated using the trial version of GraphPad Prism 10 (GraphPad Software, Boston, MA, USA).

## 3. Results

A total of 48 patients with AD dementia (10 men and 38 women) and 39 healthy controls (11 men and 28 women) in a broadly comparable age range were included in the study. Among patients with AD dementia, the duration of disease was longer than 5 years in 25.0% (n = 12), between 2 and 5 years in 35.4% (n = 17), and less than 2 years in 39.6% (n = 19). Educational status was categorized into three groups: primary school graduates or illiterate participants (64.6%), secondary/high school graduates (27.1%), and university graduates (8.3%). No statistically significant differences were observed between the AD dementia and control groups with respect to educational level. Regarding smoking status, 12.5% of the participants were current smokers, whereas 87.5% were non-smokers. Smoking status did not differ significantly between the two groups (*p* = 0.312). In contrast, the distribution of chronic comorbidities, including diabetes mellitus, hypertension, and other chronic diseases, differed significantly between the AD dementia and control groups (*p* = 0.017). Moreover, when chronic disease was analyzed as a dichotomous variable (presence vs. absence), a significant association was observed with AD dementia status (Pearson’s χ^2^(1) = 7.62, *p* = 0.006). Among patients with AD dementia, 47.9% reported a positive family history of AD dementia, whereas 52.1% had no family history. However, no statistically significant association was found between family history and AD dementia status. The demographic characteristics, clinical variables, and laboratory findings of the AD dementia and control groups, together with the corresponding statistical analyses, are summarized in Table 1.

**Figure 1:**
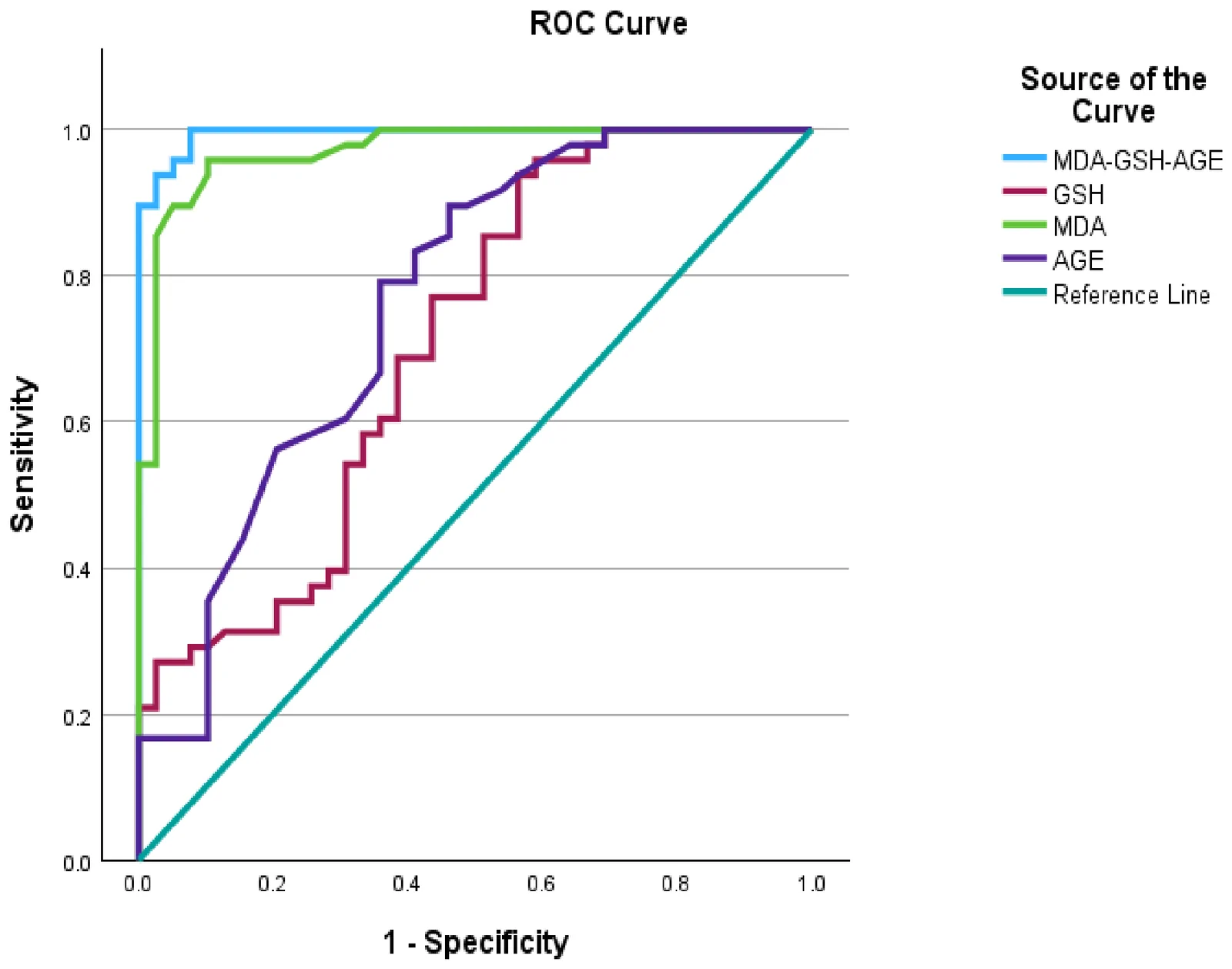
ROC curve analysis of MDA, GSH and age to predict AD dementia.

In univariate binary logistic regression analyses, MDA was positively associated with AD dementia (OR = 45.891, 95% CI: 2.588–813.989, *p* = 0.009), whereas GSH was inversely associated with AD dementia (OR = 0.789, 95% CI: 0.644–0.969, *p* = 0.024). Age was also significantly associated with AD dementia (OR = 1.235, 95% CI: 1.027–1.486, *p* = 0.025) (Table 2). A multivariable logistic regression model including MDA, GSH, and age showed excellent classification performance. The model correctly classified 36/39 controls and 48/48 AD dementia cases (overall accuracy 96.6%), corresponding to a sensitivity of 100% and a specificity of 92.3% (Table 3). ROC curve analysis demonstrated outstanding discriminative ability for the combined MDA-GSH–age model (area under the curve (AUC) = 0.995, 95% CI: 0.987–1.000, *p* < 0.001). Among individual predictors, MDA also showed excellent discrimination (AUC = 0.971, 95% CI: 0.941–1.000, *p* < 0.001), while age displayed moderate discrimination (AUC = 0.764, 95% CI: 0.663–0.867, *p* < 0.001) and GSH showed fair discrimination (AUC = 0.710, 95% CI: 0.600–0.821, *p* < 0.001) (Figure 1/Table 4).

**Figure 2:**
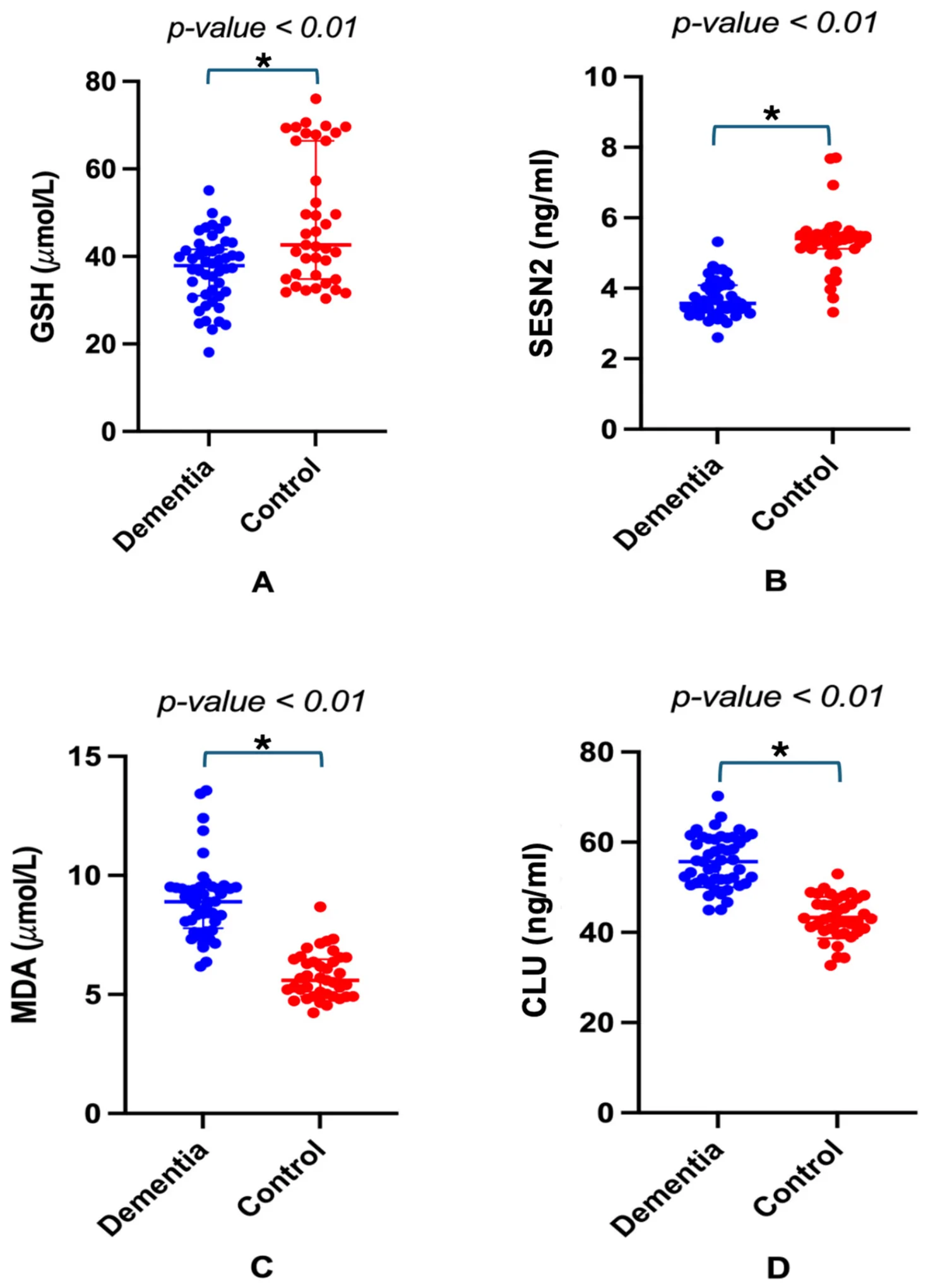
Plots of key oxidative stress parameters in AD dementia: (**A**) GSH levels in control and dementia groups; (**B**) SESN2 levels in control and dementia groups; (**C**) MDA levels in control and dementia groups; (**D**) CLU levels in control and dementia groups. * indicates a significant difference at the *p* < 0.01 level compared to the control group.

MDA levels were elevated, whereas GSH levels were reduced in patients with dementia compared with healthy controls (*p* < 0.001) (Figure 2A,C). CLU and SESN2, as biomarkers of oxidative stress, differed significantly between patients with AD dementia and healthy controls. The AD dementia group exhibited significantly lower SESN2 levels (*p* < 0.001) and significantly higher CLU levels (*p* < 0.001) compared with the control group (Figure 2B,D). Evaluation of the associations between the calculated inflammatory indices and the investigated variables in patients with AD dementia revealed no statistically significant correlations (all comparisons, *p* > 0.05). Furthermore, no significant differences were observed in lipid metabolism parameters, including LDL, HDL, and triglyceride levels (all *p* > 0.05). Similarly, fasting blood glucose, CR, hemoglobin, ferritin, vitamin B12, vitamin D, folate, and TSH levels did not differ significantly between the study groups (all *p* > 0.05).

## 4. Discussion

In this study, oxidative stress-related biochemical parameters and the emerging biomarkers CLU and SESN2 were evaluated in patients with AD dementia to investigate their potential roles in the pathogenesis of the disease. The findings demonstrated alterations in oxidative stress markers in patients with AD dementia, together with significant changes in CLU and SESN2 levels, suggesting that these molecules may be closely associated with neurodegenerative processes. In particular, the observed increase in CLU levels and decrease in SESN2 levels indicate that these biomarkers may have potential clinical utility in the diagnosis and prognosis of dementia. These findings further support the hypothesis that dysregulation of oxidative stress-related pathways contributes to the pathophysiology of AD dementia and highlight CLU and SESN2 as promising candidates for future biomarker research.

AD, the most common cause of dementia, is a complex neurodegenerative disorder characterized by progressive neuronal loss and cognitive decline. Although Aβ accumulation and tau hyperphosphorylation are widely recognized as the principal pathological hallmarks of AD, accumulating evidence indicates that oxidative stress and neuroinflammation also play pivotal roles in the initiation and progression of the disease [25]. Owing to its high oxygen consumption, abundance of polyunsaturated fatty acids, and relatively limited antioxidant defense capacity, the brain is particularly susceptible to oxidative damage. Excessive production of ROS promotes lipid peroxidation, protein oxidation, and DNA damage, ultimately leading to neuronal dysfunction and cell death. In this context, MDA, a major end product of lipid peroxidation, is widely regarded as a reliable biomarker of oxidative damage, whereas reduced GSH represents one of the principal components of the cellular antioxidant defense system. Increased MDA levels accompanied by decreased GSH concentrations have been consistently reported in patients with dementia and Alzheimer’s disease, reflecting impaired redox homeostasis and an inadequate antioxidant response [26,27]. Consistent with the established role of oxidative stress in AD, our multivariate ROC analysis demonstrated that the combination of age, serum MDA, and GSH provided excellent discrimination between patients with AD dementia and cognitively intact controls. However, given the relatively small sample size, the age difference between the groups, and the lack of independent validation, this finding should be interpreted cautiously and considered hypothesis-generating. Larger, prospective, and independently validated studies are needed to determine whether this combined approach has reproducible clinical utility.

The contribution of oxidative stress to neurodegeneration extends beyond direct cellular damage. Elevated levels of ROS also activate neuroinflammatory pathways, thereby accelerating disease progression. Consequently, increasing attention has been directed toward stress-responsive proteins that mediate cellular defense mechanisms. One such protein is CLU, also known as ApoJ, an approximately 80 kDa glycoprotein that has been identified as the second most significant genetic risk factor for late-onset AD after APOE [28,29]. CLU is involved in a wide range of biological processes, including lipid transport, regulation of the complement cascade, maintenance of cellular homeostasis, and protein quality control [12]. Under conditions of cellular stress, CLU functions as an extracellular molecular chaperone by binding to misfolded proteins, thereby preventing their aggregation and contributing to the maintenance of proteostasis [30]. Furthermore, CLU has been reported to interact with Aβ peptides, modulating amyloid fibril formation and plaque deposition. Despite these established functions, the precise role of CLU in AD remains incompletely understood. Although CLU was initially considered a neuroprotective molecule because of its cytoprotective properties, recent evidence suggests that distinct CLU isoforms and their intracellular or extracellular localization may exert divergent biological effects. In this regard, Milinkeviciute et al. demonstrated that oxidative stress alters the expression of specific CLU mRNA isoforms, thereby influencing the balance between cell survival and apoptosis [31]. Increased CLU expression has been associated with oxidative stress, neuroinflammation, and apoptotic signaling, whereas other studies have proposed that its upregulation represents a compensatory response aimed at limiting neurodegenerative damage [12,31]. Collectively, these findings suggest that alterations in CLU expression may reflect both disease severity and the magnitude of the cellular stress response. Nevertheless, the available evidence remains inconsistent. A comprehensive meta-analysis reported no significant association between plasma CLU levels and the diagnosis, risk, or severity of AD. Moreover, no significant differences in cerebrospinal fluid (CSF) CLU concentrations were observed between patients with AD and cognitively healthy controls. Based on the pooled evidence, the authors concluded that there is currently insufficient support for the use of plasma CLU as a reliable biomarker for AD [32]. The conflicting findings reported in the literature underscore the complexity of CLU biology and highlight the need for further investigation into its role in AD pathogenesis and its potential utility as a diagnostic or prognostic biomarker. In this context, the present study is expected to contribute valuable evidence by providing data from a distinct study population, thereby enriching the existing literature and improving our understanding of the relationship between CLU, oxidative stress, and AD dementia.

Another stress-responsive protein that has attracted considerable attention in neurodegenerative disorders is SESN2. Although all members of the SESN family possess potent antioxidant properties, SESN2 has been the most extensively investigated because of its cytoprotective effects against oxidative stress and cellular injury [33]. SESN2 is known to limit the accumulation of ROS, activate endogenous antioxidant defense mechanisms, and preserve cellular homeostasis through the regulation of autophagy [34,35]. Experimental evidence has demonstrated that Aβ exposure induces SESN2 expression in primary cortical neurons, leading to activation of antioxidant defense pathways and autophagic processes. Specifically, Chen et al. showed that Aβ-induced upregulation of SESN2 promoted the formation of LC3B-II, a well-established marker of autophagy, indicating that SESN2 functions as an endogenous protective mechanism against Aβ-mediated neurotoxicity [20,36]. Consistent with these findings, Faikoglu et al. demonstrated that Aβ exposure significantly increased SESN2 expression in hippocampal neurons, accompanied by alterations in autophagy-related proteins, including ATG5, Beclin-1, and LC3-II, as well as modulation of the AMPK/mTOR signaling pathway [37]. Similarly, increased SESN2 expression has been observed in transgenic animal models of AD, where its upregulation has been proposed to represent a compensatory response to heightened oxidative stress. Beyond its antioxidant activity, SESN2 has also been shown to regulate mitophagy, thereby facilitating the removal of damaged mitochondria and contributing to mitochondrial quality control [20]. Under conditions of mild-to-moderate oxidative stress, activation of SESN2 promotes cytoprotective pathways that enhance cellular survival. In contrast, prolonged or severe oxidative stress activates p53- and FOXO-mediated apoptotic signaling, ultimately leading to cell death. In this context, Budanov and Karin highlighted the critical role of SESNs as downstream effectors of p53, demonstrating their involvement in the biphasic cellular response to stress that determines the balance between survival and apoptosis [22]. Furthermore, several comprehensive review articles have emphasized that SESN2 is consistently upregulated in response to increased cellular stress during neurodegeneration, supporting its role as a key component of the endogenous defense system against oxidative injury [38,39]. Additional experimental evidence further supports the neuroprotective function of SESN2 against Aβ-induced oxidative damage. Hsieh et al. demonstrated that silencing SESN2 expression significantly increased Aβ25–35- and Aβ1–42-induced ROS generation, lipid peroxidation, and levels of 8-hydroxy-2′-deoxyguanosine (8-OH-dG), a well-established biomarker of oxidative DNA damage. Conversely, overexpression of either the N-terminal or C-terminal domain of SESN2 markedly attenuated Aβ25–35-induced ROS production [40]. Collectively, these findings suggest that alterations in SESN2 expression may provide valuable insight into the oxidative burden and adaptive cellular responses associated with neurodegenerative diseases, highlighting its potential as a biomarker and therapeutic target in dementia.

In conclusion, the complex interplay among oxidative stress, antioxidant defense mechanisms, and cellular stress-response proteins appears to play a pivotal role in the pathogenesis of AD dementia. The combined evaluation of MDA, reduced GSH, CLU, and SESN2 in the present study may contribute to a more comprehensive understanding of the molecular mechanisms underlying neurodegeneration. In particular, the observed increase in CLU levels together with the decrease in SESN2 levels suggests that these molecules may serve as promising biomarkers reflecting the link between oxidative stress and neurodegenerative processes. Nevertheless, larger prospective studies with well-characterized patient populations are required to clarify their precise roles in disease progression and to establish their clinical utility as diagnostic and prognostic biomarkers. In the present study, no statistically significant associations were identified between the calculated inflammatory indices and the investigated biochemical parameters in patients with AD dementia. This finding suggests that systemic inflammatory indices may not consistently possess sufficient sensitivity to reflect the clinical characteristics of AD dementia or the molecular alterations evaluated in this study. Furthermore, the heterogeneous nature of the study population, the relatively limited sample size, and the potential influence of confounding factors may also have contributed to the lack of significant associations. Future large-scale, longitudinal studies incorporating comprehensive inflammatory and oxidative stress profiling are warranted to further elucidate the interplay between systemic inflammation, oxidative stress, and neurodegeneration in AD dementia.

Despite the novel findings of the present study, several limitations should be acknowledged. First, the relatively small sample size and the unequal distribution of male and female participants may have limited the statistical power of sex-specific analyses and the generalizability of the findings across sexes. Second, although AD dementia severity was classified into mild, moderate, and severe stages, the number of participants within each subgroup was insufficient to allow robust statistical comparisons. This limitation was primarily related to the difficulty in recruiting a larger number of clinically diagnosed AD dementia patients. Therefore, future studies conducted in larger cohorts are needed to validate the present findings across different stages of disease severity. Another important limitation is that, although oxidative stress markers and related biomarkers were evaluated in patients with AD dementia, mediation analyses aimed at elucidating the relative contributions and potential mechanistic interactions of these parameters in AD dementia pathophysiology could not be performed. This limitation mainly stems from the cross-sectional design and the limited sample size of the study, as mediation analyses require larger populations and more robust multivariable modeling approaches. Furthermore, the cross-sectional nature of the study precludes the establishment of causal relationships between oxidative stress, CLU, SESN2, and dementia progression. Consequently, large-scale prospective and longitudinal studies are warranted to clarify the comparative roles of oxidative stress-related biomarkers and to further elucidate their contribution to the development and progression of AD dementia.

## 5. Conclusions

In conclusion, the findings of the present study demonstrate a significant association between AD dementia and increased oxidative stress, as evidenced by elevated MDA levels and reduced GSH concentrations. These results support the concept that disruption of redox homeostasis is closely involved in the pathophysiology of AD dementia. Furthermore, the observed increase in CLU levels and decrease in SESN2 levels suggest that alterations in cellular stress responses and antioxidant defense mechanisms may contribute to the neurodegenerative processes underlying AD dementia. Collectively, our findings highlight the potential involvement of oxidative stress-related pathways in the pathogenesis of AD dementia and suggest that these alterations may be associated with disease-related metabolic disturbances. The combined assessment of CLU and SESN2, together with conventional oxidative stress biomarkers, may provide valuable insights into the molecular mechanisms of AD dementia and support their potential utility as candidate diagnostic biomarkers. However, larger, well-designed prospective studies are warranted to validate these findings, clarify the precise biological roles of these biomarkers, and further elucidate the contribution of oxidative stress to the development and progression of AD dementia.

## 6. Limitations

This study has several limitations that should be considered when interpreting the findings. First, the study groups were not fully comparable in terms of age, and although age was taken into account in the statistical analyses, residual confounding by age cannot be completely excluded. Second, dietary habits and the use of vitamins or nutritional supplements were not systematically assessed, and information regarding medications and treatments was not uniformly available. Therefore, the potential effects of these factors on the investigated biomarkers cannot be fully excluded. Third, the presence of comorbidities differed between the study groups and may have influenced MDA, GSH, CLU, SESN2, and inflammatory marker levels, representing a potential source of residual confounding. In addition, the relatively small sample size and the use of multiple comparisons, regression analyses, and ROC analysis may have limited the statistical power and generalizability of the findings and may increase the possibility of chance findings. Finally, although MDA and GSH were selected for the regression and ROC analyses based on their representation of oxidative stress and antioxidant status, respectively, the diagnostic analyses should be interpreted cautiously. Larger, prospective studies with well-matched groups, systematic assessment of dietary and treatment-related factors, and comprehensive adjustment for potential confounders are warranted to validate these findings.

## Figures and Tables

**Table 1:** Demographics and laboratory findings of control and AD dementia groups.

Demographic Data	Control n = 39	Dementia n = 48	*p*-Value
Age	67 (59–85)	78.5 (65–89)	0.061
Male, sex	11 (28.21%)	10 (20.84%)	0.424
Female, sex	28 (71.8%)	38 (79.17%)	
Weight (kg)	72.42 ± 12.64	70.38 ± 11.35	0.431
Height (m)	1.68 ± 0.1	1.63 ± 0.09 *	0.004
BMI (Body Mass Index) (kg/m^2^)	25.72 (18.43–34.16)	25.89 (19.26–39.96)	0.359
Smoking (no)	31 (79.49%)	42 (87.5%)	0.312
Smoking (yes)	8 (20.52%)	6 (12.5%)	
Pre-High-School Graduate	26 (66.67%)	35 (72.92%)	0.527
High School and University Graduate	13 (33.34%)	13 (27.09%)	
Diabetes Mellitus	13 (27.1%)	4 (10.3%) *	0.017
Hypertension	15 (31.3%)	13 (33.3%) *	
Other Diseases	12 (25%)	5 (12.8%) *	
**Laboratory Findings**	**Control n = 39**	**Dementia n = 48**	***p*-Value**
Fasting Blood Glucose (mg/dL)	97 (76–175)	101.5 (70–281)	0.106
Urea (mg/dL)	25.47 (10–80)	38.16 (15–81.53)	<0.001 *
Hemoglobin (g/dL)	13.64 ± 1.66	13.02 ± 1.43	0.064
Vitamin B12 (pg/mL)	411.1 (181–1533)	457.5 (196–1479)	0.173
Vitamin D (µg/L)	25.38 (3–120)	22.61 (1–80.39)	0.481
Folic Acid (ng/mL)	7.54 (3.2–15.45)	7.05 (2.47–20)	0.558
HDL (mg/dL)	53.22 ± 13.72	50.89 ± 12.86	0.416
LDL (mg/dL)	124.48 ± 44.32	124.65 ± 38.91	0.984
Triglyceride (mg/dL)	136 (38–321)	135.5 (57–383)	0.698
CRP (mg/L)	1.41 (0.2–28.27)	2.18 (0.38–150.36)	0.060
Ferritin (ug/L)	47.26 (4.09–278)	74.46 (5–320.8)	0.150
WBC (10^3^/mm^3^)	6.47 (4.21–13.22)	7.19 (3.33–15.06)	0.290
Neutrophil (10^9^/L)	3.77 (2.04–8.61)	4.34 (2.14–61.1)	0.101
Lymphocyte (10^3^/µL)	2.07 (0.95–3.97)	2.08 (0.6–28.7)	0.798
Monocyte (10^3^/µL)	0.54 (0.31–1.15)	0.55 (0.33–7.5)	0.617
Platelet (10^3^/µL)	262 (186–532)	245.5 (84–389)	0.085
ALT (U/L)	15 (6–51)	11.7 (5–48.9)	0.034 *
AST (U/L)	17 (8–40)	18 (8–49)	0.735
GGT (U/L)	15 (10–79)	15.5 (8–120)	0.871
CR (mg/dL)	0.8 (0.56–2.25)	0.84 (0.58–2.41)	0.570
e-GFR	76 (19.18–131.69)	68.42 (23–93)	0.051
TSH (mIU/L)	1.95 (0.58–15.65)	1.69 (0.44–23.74)	0.169
MDA (µmol/L)	5.6 (4.23–8.69)	8.9 (6.18–13.58)	<0.001 *
GSH (µmol/L)	42.63 (30.39–76.03)	37.9 (18.07–55.11)	0.001 *
CLU (ng/mL)	43.31 ± 4.62	55.73 ± 5.73	<0.001 *
SESN2 (ng/mL)	5.41 (3.33–7.7)	3.57 (2.61–5.32)	<0.001 *
SII	352.46 (158.23–1083.91)	388 (26.55–1260.72)	0.107
AISI	183.96 (88.91–672.02)	211.61 (13.54–2797.74)	0.107
SIRI	1.06 (0.51–3.86)	1.22 (0.08–16.08)	0.160
LMR	3.47 (1.76–6.6)	3.75 (1.25–56.27)	0.868
PLR	84.06 (43.83–183.16)	92.55 (6.06–290)	0.710
NLR	2.03 (0.91–6.23)	2.15 (0.15–6.12)	0.196
dNLR	1.42 (0.71–3.69)	1.49 (−1.18–23.06)	0.196
LCR	1.29 (0.05–15.45)	0.86 (0.02–30.65)	0.165

Note: Continuous data are presented as median (min–max) or mean ± standard deviation, as appropriate. Categorical variables are presented as number (percentage). A *p*-value of < 0.05 was considered statistically significant. Statistically significant results are presented in bold. * Statistical difference with the control group *p* < 0.05. Continuous variables were compared using the independent-samples *t*-test or Mann–Whitney U test, and categorical variables using the Pearson chi-square test.

**Table 2:** Univariate binary logistic regression analyses of parameters associated with AD dementia.

	*p*-Value	OR	95% CI for OR
**MDA**	0.009	45.891	2.588–813.989
**GSH**	0.024	0.789	0.644–0.969
**Age**	0.025	1.235	1.027–1.486

**Table 3:** Classification report of logistic regression (MDA-GSH–age) classifier.

Observed	Predicted	
	Control	Dementia
**Control**	36	3
**Dementia**	0	48

**Table 4:** Classification report of logistic regression ROC curve.

Test Result Variable(s)	MDA-GSH–Age	MDA	GSH	Age
**Area under the curve**	0.995	0.971	0.710	0.764
***p*** **-Value**	0.000	0.000	0.000	0.000
**95% CI**	0.987–1.000	0.941–1.000	0.6–0.821	0.663–0.867
**Sensitivity**	1.000	0.958	0.938	0.896
**Specificity**	0.923	0.897	0.436	0.538
**Precision**	0.941	0.920	0.672	0.705
**Recall**	1.000	0.958	0.938	0.896

## Data Availability

Data supporting the findings of this study are included within the article. Should raw data files be needed in another format, they are available from the corresponding author.
